# Improving the Quality of Life of Parkinson’s Disease Patients in Cambodia: A Perspective on Addressing the Gaps

**DOI:** 10.3389/phrs.2026.1609756

**Published:** 2026-04-14

**Authors:** Virak Sorn

**Affiliations:** Faculty of Health Sciences and Biotechnology, University of Puthisastra, Phnom Penh, Cambodia

**Keywords:** Cambodia, health systems strengthening, non-communicable diseases, Parkinson’s disease, quality of life

## Introduction

Parkinson’s disease (PD) is the second most common neurodegenerative disorder worldwide and primarily affects the elderly. Longitudinal estimates show PD patients have increased from 3.15 million in 1990 to over 11.77 million in 2021, representing a 274% increase [1]. This indicates that the global burden of PD has significantly increased. This escalation is mirrored in mortality and morbidity metrics, with PD-related deaths and disability-adjusted life years rising by over 100% and 81%, respectively [2]. These trends are particularly pronounced in low- and middle-income countries (LMICs) undergoing demographic transitions, with global projections suggesting a total of 25.2 million cases by 2050 [3].

Cambodia, with a population of 17.85 million, faces an age-standardized prevalence rate of PD being 88 to 94 per 100,000 population and is experiencing rapid population aging and an increasing burden of non-communicable diseases (NCDs) [1, 2]. Although the Ministry of Health (MOH) has implemented reforms to expand primary healthcare and financial protection, neurological disorders such as PD remain largely under-prioritized within current national NCD strategies [4, 5]. The absence of comprehensive national epidemiological data masks a critical need for proactive health planning to prevent widening inequities in access to specialized care across both urban and rural areas [6, 7].

Comprehensive management of PD necessitates a multidisciplinary framework that encompasses neurologists, specialized medications, surgical options, and rehabilitation essential to manage symptoms like tremors, stiffness, and slowness. Yet, in many LMIC settings, including Cambodia, access to such comprehensive care remains limited.

## Current Gaps in Parkinson’s Disease Care in Cambodia

### Limited Specialist Availability and Centralized Services

Gaps in care of PD patients that are unique to Cambodia include a lack of a public-owned central neurological institute with medical, surgical, rehabilitative, research, and telemedicine facilities. Neurological services in Cambodia are concentrated in the capital city and a few major urban centers. Movement disorder specialists are scarce, and most PD patients are managed by general practitioners or internists with limited specialized training in movement disorders [7].

Evidence from high-income settings shows that care under neurologists is associated with improved outcomes, including fewer hospitalizations and better survival [8]. In Cambodia, however, geographic and financial barriers restrict access to specialist care. Patients from rural provinces often incur substantial transportation and accommodation costs, resulting in delayed diagnosis and inconsistent follow-up [6].

### Financial Barriers and FragmentedCoverage

Although Cambodia has expanded health equity funds and social health protection schemes, however, coverage for chronic neurological conditions remains limited [7]. Long-term pharmacotherapy, periodic monitoring, and potential surgical interventions are financially burdensome for many households. Out-of-pocket expenditures remain significant in the private sector, where many patients seek care due to perceived quality differences [5]. This creates inequitable access and contributes to treatment discontinuity, worsening symptom control and quality of life (QoL).

### Inadequate Rehabilitation and Multidisciplinary Care

Multidisciplinary care, such as access to neurologists, medications, surgical treatment, and rehabilitation, is necessary for the best management where it can reduce the symptoms of PD, allowing patients to live a meaningful life. In Cambodia, such services are limited and often in the capital and urban-based. Although community-based rehabilitation programs exist in Cambodia for individuals with disabilities broadly, there is still very little integration for PD. Without structured rehabilitation, patients experience progressive loss of independence, social withdrawal, and caregiver burden.

### Social Isolation and Caregiver Burden

Beyond clinical challenges, PD patients in Cambodia often face substantial social and psychological difficulties. Traditionally, Cambodian families play a central role in caring for elderly relatives. However, rapid urbanization and labor migration have altered family structures. Many younger adults move to cities or abroad for employment, leaving older family members with reduced social support.

PD patients often experience stigma due to visible motor symptoms. Care-seeking is delayed by misconceptions that sometimes attribute symptoms to spiritual or normal aging. Caregivers, frequently elderly spouses, face physical, emotional, and financial strain without formal support systems. Improving QoL, therefore, requires addressing psychosocial dimensions alongside clinical treatment.

## A Way Forward: Strategic Priorities for Cambodia

### Establishing a National Neurological Coordination Framework

The MOH should establish a national coordination framework for neurology services. By designating a central referral hub in the capital to develop clinical guidelines and provide advanced care, provincial hospitals could function as satellite centers for the management of movement disorders. This central hub would (1) provide specialist consultations and advanced clinical management; (2) develop and disseminate national clinical guidelines for PD; (3) coordinate training and capacity-building programs for provincial clinicians; and (4) support research, data collection, and disease surveillance.

Such a coordinated model would facilitate the systematic transfer of specialized knowledge to lower levels of the health system, ensuring that district-level services are strengthened through structured referral pathways and regular specialist outreach visits.

### Integrating PD Care Into NCD and UHC Frameworks

PD should be explicitly incorporated into Cambodia’s national strategies for NCDs and included within essential health service packages. This integration should encompass several key measures. First, the consistent availability of essential medications for PD management should be ensured across public health facilities. Second, financial protection mechanisms should be expanded to cover long-term neurological care, thereby reducing the economic burden on patients and their families. Third, PD-related indicators should be incorporated into the national health information system to enable systematic monitoring, planning, and evaluation of services.

Aligning PD services with the broader objectives of universal health coverage would contribute to more equitable access to care while promoting the long-term sustainability of neurological health services in Cambodia.

### Leveraging Telemedicine and Digital Health

Telemedicine represents a practical and cost-effective approach to addressing Cambodia’s shortage of neurological specialists. With the increasing penetration of mobile phones and internet connectivity—even in rural areas—telehealth platforms can facilitate remote consultations, reduce the need for long-distance travel, and enhance continuity of care for PD patients [5]. Telemedicine can serve key functions such as follow-up consultations and medication adjustments, remote monitoring of symptom progression and treatment response, and virtual rehabilitation services by guided physiotherapy or speech therapy.

A blended care model—combining periodic in-person clinical visits with teleconsultations—may offer an effective strategy to optimize clinical outcomes while minimizing financial and logistical burdens for patients and healthcare systems.

### Strengthening Rehabilitation and Community-Based Interventions

Rehabilitation services should be integrated into provincial and district hospital systems to improve access to comprehensive care for PD patients. A pragmatic initial step would involve training existing physiotherapists and community health workers in PD-specific rehabilitation protocols, thereby strengthening local capacity without requiring substantial new infrastructure.

Community-based exercise and movement programs—adapted to local cultural contexts—could further support improvements in motor function, physical activity, and social engagement among individuals living with PD. Group-based activities, such as structured movement programs or dance therapy, have demonstrated benefits for both motor and non-motor symptoms in various international settings [9] and could be piloted in urban centers before gradual expansion to provincial areas.

Integrating these rehabilitation initiatives with existing disability support services and elderly care programs would help minimize service duplication while enhancing program reach and sustainability.

### Capacity Building and Public Awareness

Achieving sustainable improvements in PD care requires long-term investments in workforce development, public awareness, and caregiver support. Strengthening the capacity of general practitioners to recognize early symptoms of PD and initiate appropriate management is essential for promoting timely diagnosis and intervention.

Public awareness campaigns are also needed to reduce stigma associated with neurological disorders and to encourage individuals experiencing early symptoms to seek medical consultation. In parallel, caregiver education programs should be implemented to provide practical guidance on symptom management, fall prevention, and the psychological and emotional aspects of long-term care.

### Policy Implications and Research Needs

Cambodia lacks robust epidemiological data on PD prevalence, incidence, and economic burden. National-level surveillance and operational research are urgently needed to inform resource allocation. Collaborative research partnerships—regional and international—could support capacity building, guideline development, and evaluation of telemedicine and rehabilitation interventions [10]. Importantly, PD care should be viewed not solely as a clinical issue but as a public health priority within the broader aging and NCD agenda.

### Conclusion

PD poses a growing challenge to Cambodia’s health system. Current gaps—limited specialist access, financial barriers, fragmented rehabilitation, and inadequate community support—significantly impair QoL for patients and caregivers. A coordinated, multi-level strategy integrating specialist services, primary care strengthening, telemedicine expansion, rehabilitation, financial protection, and public awareness can transform PD care in Cambodia. With strategic policy commitment and health-system decentralization, Cambodia can proactively address this emerging burden and ensure that individuals living with PD maintain dignity, independence, and social participation.
